# Deletion of *Nlrp3* protects from inflammation-induced skeletal muscle atrophy

**DOI:** 10.1186/s40635-016-0115-0

**Published:** 2017-01-17

**Authors:** Nora Huang, Melanie Kny, Fabian Riediger, Katharina Busch, Sibylle Schmidt, Friedrich C. Luft, Hortense Slevogt, Jens Fielitz

**Affiliations:** 1 Experimental and Clinical Research Center (ECRC), Charité-Universitätsmedizin Berlin, Max Delbrück Center (MDC) for Molecular Medicine in the Helmholtz Association, Berlin, Germany; 2 Department of Cardiology, Heart Center Brandenburg and Medical University Brandenburg (MHB), Bernau, Germany; 3 Department of Cardiology and Pneumology, Medical University Brandenburg (MHB), Brandenburg, Germany; 4 Berlin Institute of Health (BIH), Kapelle-Ufer 2, 10117 Berlin, Germany; 5 ZIK Septomics, Host Septomics, Jena, Germany; 6 Integrated Research and Treatment Center—Center for Sepsis Control and Care (CSCC), Jena University Hospital, Jena, Germany

**Keywords:** Sepsis, IL-1β, Muscle weakness, ICUAW

## Abstract

**Background:**

Critically ill patients develop atrophic muscle failure, which increases morbidity and mortality. Interleukin-1β (IL-1β) is activated early in sepsis. Whether IL-1β acts directly on muscle cells and whether its inhibition prevents atrophy is unknown. We aimed to investigate if IL-1β activation via the Nlrp3 inflammasome is involved in inflammation-induced atrophy.

**Methods:**

We performed an experimental study and prospective animal trial. The effect of IL-1β on differentiated C2C12 muscle cells was investigated by analyzing gene-and-protein expression, and atrophy response. Polymicrobial sepsis was induced by cecum ligation and puncture surgery in Nlrp3 knockout and wild type mice. Skeletal muscle morphology, gene and protein expression, and atrophy markers were used to analyze the atrophy response. Immunostaining and reporter-gene assays showed that IL-1β signaling is contained and active in myocytes.

**Results:**

Immunostaining and reporter gene assays showed that IL-1β signaling is contained and active in myocytes. IL-1β increased *Il6* and *atrogene* gene expression resulting in myocyte atrophy. *Nlrp3* knockout mice showed reduced IL-1β serum levels in sepsis. As determined by muscle morphology, organ weights, gene expression, and protein content, muscle atrophy was attenuated in septic *Nlrp3* knockout mice, compared to septic wild-type mice 96 h after surgery.

**Conclusions:**

IL-1β directly acts on myocytes to cause atrophy in sepsis. Inhibition of IL-1β activation by targeting Nlrp3 could be useful to prevent inflammation-induced muscle failure in critically ill patients.

**Electronic supplementary material:**

The online version of this article (doi:10.1186/s40635-016-0115-0) contains supplementary material, which is available to authorized users.

## Background

A major contributor of intensive care unit (ICU)-acquired weakness (ICUAW) is a severe and disabling muscle atrophy leading to loss in strength and mass [1–4]. ICUAW is associated with increased morbidity and mortality and has a significant impact on healthcare systems [5, 6]. Sepsis and systemic inflammation are major risk factors for ICUAW [7, 8]. Importantly, inflammation and acute-phase response occur early and directly in muscle and affect disease progression in ICUAW. Recently, others and we reported an imbalanced protein homeostasis caused by increased protein degradation and reduced protein synthesis in the skeletal muscle [4, 9–11]. Muscular-motor protein breakdown, especially myosin heavy chain (MyHC), via the protein degrading ubiquitin proteasome system (UPS), is a prominent feature of muscle atrophy [1, 4, 10–13]. The E3 ligase, muscle RING finger (MuRF) 1 (*TRIM63*), and F-box protein atrogin 1 (*FBXO32*) are increased in muscle during atrophy and mediate degradation of structural proteins [4, 10]. Interleukin-1β (IL-1β) is one of the most activated cytokines in sepsis [14–20]. In muscle, IL-1β increases MuRF1 and atrogin 1 expression implicating a function in atrophy [21–23]. However, if IL-1β directly causes muscle atrophy in sepsis and if inhibition of IL-1β prevents this response is unknown. IL-1β production and secretion requires three consecutive steps that are tightly controlled, namely expression, cleavage, and secretion. Whereas inflammatory cytokines increase expression of pro-IL-1β, which is the inactive proform of IL-1β, its conversion to IL-1β, and its secretion is mediated by caspase-1 activating inflammasomes [14, 24–28]. Inflammasomes are multi-protein complexes of the innate immune system [29] and involved in the pathogenesis of sepsis [30]. Cytoplasmic receptors of the nucleotide binding domain (NOD)-like receptor (NLR) family are key components of the inflammasome, of which the best characterized NLR is NLRP3 [31, 32]. The NLRP3 inflammasome regulates maturation and secretion of IL-1β [32]. IL-1β signal transduction occurs via the IL-1 receptor, which is associated with IL-1 receptor-associated kinase 1 (IRAK1) that activates the transcription factor nuclear factor-kappa B (NF-κB) [33]. NLRP3 is contained in muscle, and its activity is increased in myopathies [34]. However, the function of NLRP3 and IL-1β in ICUAW is unknown. We tested the hypothesis that IL-1β, depending on the Nlrp3 inflammasome, contributes to inflammation-induced atrophy in vitro and in vivo.

## Methods

### Animal model

Animal procedures were performed in accordance with the guidelines of the Max-Delbrück Center for Molecular Medicine, were approved by the Landesamt für Gesundheit und Soziales, Berlin, Germany (G207/13, G129/12), and followed the “Principles of Laboratory Animal Care” (NIH publication No. 86-23, revised 1985) and the current version of German Law on the Protection of Animals. *Nlrp3* knockout (KO) mice were kindly provided by Aubry Tardivel and Nicolas Fasel (University of Lausanne) [35]. Cecal ligation and puncture (CLP) surgery was performed to induce polymicrobial sepsis in 12- to 16-week-old male *Nlrp3* KO or wild-type (WT) mice as recently described [36–38]. Sham mice were treated identically except for the ligation and puncture of the cecum. Mice were sacrificed 96 h after surgery. For more information, see Additional file 1.

### Molecular and cell biology analysis

For detailed information about quantitative RT-PCR (qRT-PCR), western blotting, immunostaining, and cell culture, see Additional file 1. Measurements of serum IL-1β were performed by using the Mouse ELISA Kit for IL-1β (Abcam, ab100704) according to the manufacturers’ protocol.

### Statistical tests

All experiments were performed independently and at least three times using biological triplicates each. All qRT-PCR gene expression data from mouse and cell culture samples was analyzed by one-way ANOVA with post hoc correction (Tukey’s post-comparison test). Paired *t* test was used to study the distribution of myotube diameter in C2C12 myotubes. Survival curves were compared with a Mantel-Cox test. Differences were considered statistically significant at *p* < 0.05. Data are shown as mean ± standard error of the mean (SEM) in bar plots. Plots and statistics calculation were done by using the GraphPad Prism® 6 program (GraphPad Software, La Jolla, CA, USA), Adobe Illustrator CS6, version 16.0.0, and Photoshop CS6, version 13.0. The documentation of immunofluorescence and histological staining results was performed with a Leica fluorescence microscope using Leica cameras (DFC 360 FX and DFC 425) and the LAS.AF software (version: 2.4.1 build 6384 and the LAS3.1 software (version 2.5.0.6735).

## Results

### IL-1β induces myocyte atrophy in vitro

Recently, we showed that inflammation and acute-phase response participates in the pathogenesis of ICUAW in patients [10]. However, whether IL-1β is synthesized in muscle and whether Nlrp3-mediated IL-1β maturation is involved in inflammation-induced atrophy was unknown. We performed qRT-PCR to investigate if sepsis increases *Il1b* or *Nlrp3* expression in *gastrocnemius/plantaris* or *tibialis anterior* muscle of mice and found that sepsis induced *Il1b* and *Nlrp3* expression in both muscles (Additional file 2A, B). *Il6* expression was also induced. These data indicate that IL-1β and Nlrp3 are contained and activated in muscles during sepsis.

**Fig. 1:**
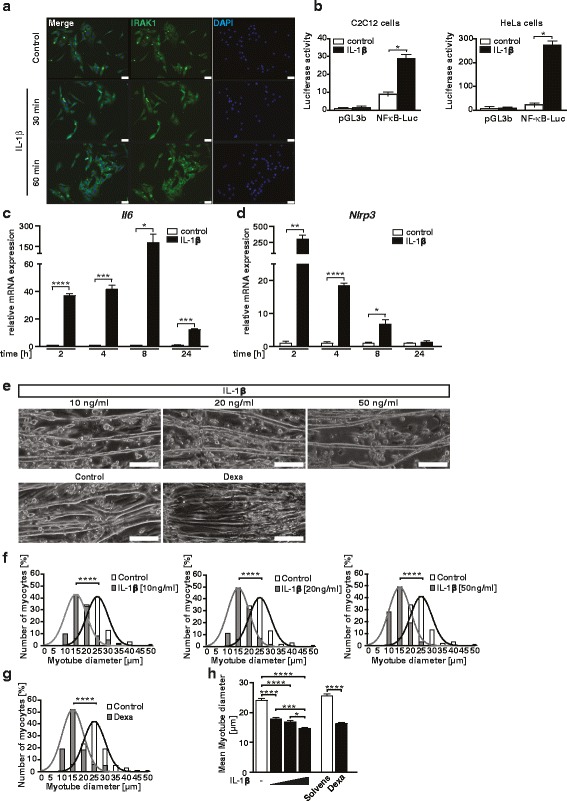
The IL-1β signaling pathway is contained and active in C2C12 myocytes. **a** C2C12 muscle cells were treated with human recombinant IL-1β (10 ng/ml) or vehicle for 30 min and 1 h. Immunocytochemistry with anti-IRAK1 antibody shows cytoplasmic-to-nuclear translocation of IRAK1 in response to IL-1β after 30 min. Nuclei were stained in blue (DAPI). *Scale bar* = 50 μm. **b** Synthetic luciferase reporters with multimerized NF-κB sites (NF-κB-Luc) were transfected into C2C12 (**b**, *left panel*) and HeLa (**b**, *right panel*) cells, together with LacZ as transfection control. Cells were treated with recombinant IL-1β (10 ng/ml) for 24 h. *n* = 3. **c**, **d** C2C12 cells were differentiated for 8 days and treated with human recombinant IL-1β (10 ng/ml) for different time points as indicated. qRT-PCR analysis of *Il6* and *Nlrp3*. mRNA expression was normalized to *Gapdh*. All data are reported as fold change ± SEM. **e–h** IL-1β increases *Nlrp3* expression and induces atrophy in differentiated C2C12 myocytes in vitro. C2C12 cells were differentiated for 8 days and treated with human recombinant IL-1β (10, 20, and 50 ng/ml) for 72 h. Dexamethasone (10 μM/ml) treatment was used as atrophy control. **e** Representative light microscopy pictures. *Scale bar* = 250 μm. **f**, **g** Frequency distribution histograms of cell width of IL-1β (10, 20, and 50 ng/ml) and dexamethasone-treated myotubes, as indicated, compared to vehicle-treated myotubes, *n* = 100 cells per condition. **h** Mean myotube width. Data are presented as mean ± SEM. *p < 0.05, **p < 0.01, ***p < 0.001, ****p ≤ 0.0001

To investigate if the IL-1β signaling pathway is contained and active in myocytes, we analyzed cytoplasmic-to-nuclear translocation of IL-1 receptor type I (IL-1R1) associated kinase 1 (IRAK-1) in C2C12 muscle cells. C2C12 myoblasts were originally isolated from wild-type mice [39] and selected for its ability to differentiate to myotubes expressing characteristic muscle proteins [40]. Others [41–43] and we [36, 38, 44] have used this cell line earlier to investigate mechanisms of inflammation-induced myocyte atrophy. Using immunocytochemistry, we found that 30 min of IL-1β treatment resulted in an increased cytoplasmic-to-nuclear shift of IRAK-1 in C2C12 myocytes (Fig. 1a) indicating that the IL-1β pathway is active in myocytes. Since IL-1β mediates its effects via NF-κB in non-myocytes, a luciferase reporter assay was used to test if this response also occurs in myocytes. The same assay performed in HeLa cells was used as positive control. IL-1β treatment induced the NF-κB promoter in muscle and non-muscle cells (Fig. 1b), indicating that IL-1β activates NF-κB dependent signaling events in muscle cells. To test if IL-1β induces its target genes in myocytes, we treated C2C12 myotubes with recombinant IL-1β for different time points and quantitated *Il6* expression (Fig. 1c). IL-1β induced *Il6* and *Nlrp3* expression in myocytes after 2 h of treatment (Fig. 1c, d). Together, these data indicate that the IL-1β pathway is functional in myocytes. To investigate if IL-1β induces myocyte atrophy, we treated C2C12 myotubes with increasing amounts of recombinant IL-1β and vehicle, respectively, for 72 h and measured myotube diameters. IL-1β treatment caused a significant reduction of myotube diameters after 72 h (Fig. 1e). Frequency distribution histograms of myotube diameters showed a dose-dependent increase in the number of thinner myotubes resulting in a leftward shift of the histogram and a dose-dependent decrease in mean myotube diameters after 72 h of treatment (Fig. 1f, h). Dexamethasone (Dexa), which was used as positive control, resulted in myotube atrophy after 72 h (Fig. 1e, g, h).

**Fig. 2:**
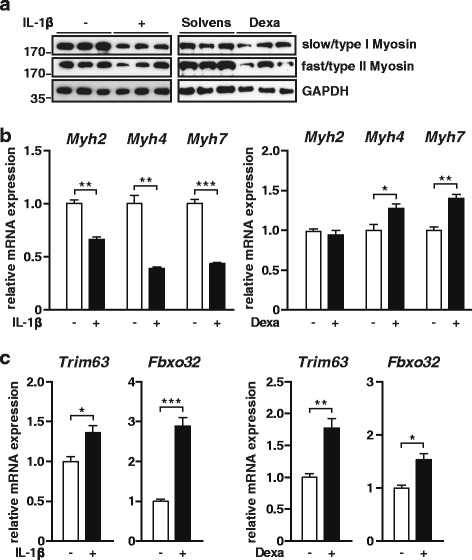
IL-1β treatment induces *Trim63* (MuRF1) *and Fbxo32* (atrogin 1) gene expression and reduces slow and fast myosin heavy chain (MyHC) in C2C12 myotubes. **a** C2C12 cells were differentiated for 8 days and treated with human recombinant IL-1β (10 ng/ml) for 72 h. Dexamethasone (10 μM/ml) treatment was used as atrophy control. Western blot analysis with anti-myosin heavy chain (MyHC) slow and anti-MyHC-fast antibody. *n* = 3. GAPDH was used as loading control. **b** C2C12 cells were differentiated for 8 days and treated with human recombinant IL-1β (10 ng/ml) for 24 h. Dexamethasone (10 μM/ml) treatment was used as atrophy control. qRT-PCR analysis of myosin heavy chain (*Myh*) 2, *Myh4*, and *Myh7* expression. mRNA expression was normalized to *Gapdh*. Data are presented as mean ± SEM. *n* = 3. *p ≤ 0.05; **p ≤ 0.01; ***p ≤ 0.001. **c** C2C12 cells were differentiated for 8 days and treated with human recombinant IL-1β (10 ng/ml) for 2 h. Dexamethasone (10 μM/ml) treatment was used as atrophy control. qRT-PCR analysis of *Trim63* (MuRF1) and *Fbxo32* (atrogin 1) expression. mRNA expression was normalized to *Gapdh*. Data are presented as mean ± SEM. *n* = 3. *p ≤ 0.05; **p ≤ 0.01; ***p ≤ 0.001

Because a reduction in MyHC proteins is consistently observed in inflammation-induced atrophy [1, 10], we investigated if IL-1β causes a reduction in MyHC protein. C2C12 myotubes were treated with recombinant IL-1β and vehicle, respectively, for 72 h and Western blot was performed. Indeed, IL-1β decreased fast and slow MyHC protein contents (Fig. 2a). As expected, Dexa treatment caused a reduction in slow and fast MyHC contents after 72 h (Fig. 2a). Recently, we reported that inflammation-induced atrophy is caused by a dysregulation in protein homeostasis with decreased *MyHC* expression and increased UPS-dependent MyHC degradation [10]. Therefore, we investigated if IL-1β causes a reduction in *MyHC* expression. C2C12 myotubes were treated with IL-1β and vehicle, respectively, for 24 h and *Myh2*, *4*, and *7* expression, encoding fast/type IIa, fast/type IIb and slow/type I MyHC, respectively, was quantitated by qRT-PCR (Fig. 2b). IL-1β treatment led to a decreased *Myh2*, *Myh4*, and *Myh7* expression after 24 h; whereas Dexa led to an increased *Myh4* and *Myh7* but not *Myh2* expression (Fig. 2b). To test if IL-1β activates atrophy gene expression involved in MyHC degradation, we treated C2C12 myotubes with IL-1β for 2 h and quantitated *Trim63* and *Fbxo32* expression by qRT-PCR. IL-1β significantly increased *Trim63* and *Fbxo32* expression, indicating that MuRF1 and atrogin 1 are involved in IL-1β-induced atrophy. Likewise, Dexa treatment increased *Trim63* and *Fbxo32* expression in myocytes (Fig. 2c). These data indicate that IL-1β causes a disturbed protein homeostasis contributing to IL-1β mediated atrophy.

### *Nlrp3* KO mice are protected against inflammation-induced atrophy

**Fig. 3:**
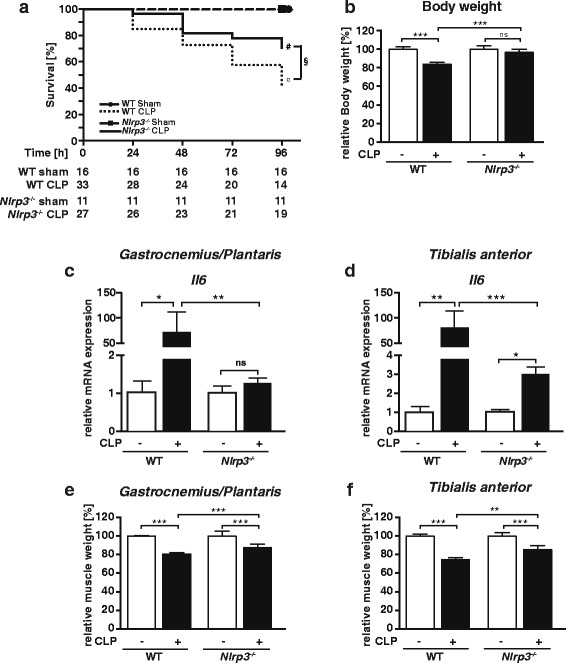
*Nlrp3* KO mice have a survival benefit and do not lose body or muscle weight during 96 h of sepsis. Twelve- to 16-week-old male *Nlrp3* KO and WT mice were subjected to CLP or sham surgery. **a** Survival *curves* show an improved survival of *Nlrp3* KO compared to WT mice following CLP surgery. All sham mice survived to the experimental end point. Survival analysis was performed with Log-Rank-test; WT sham vs. WT CLP: *p* ≤ 0.001, (*circle*); *Nlrp3* KO sham vs. *Nlrp3* KO CLP: *p* ≤ 0.05, (*number sign*); WT CLP vs. *Nlrp3* KO CLP: *p* ≤ 0.05 (*section sign*). The numbers of animals in each experimental group are indicated in the figure. **b** Body weight at 96 h after surgery. CLP-treated *Nlrp3* KO (*n* = 16); sham *Nlrp3* KO (*n* = 8), WT CLP (*n* = 12), WT sham (*n* = 13). **c**, **d** qRT-PCR analysis of *Il6* expression in *gastrocnemius/plantaris* and *tibialis anterior* muscles of WT sham (*n* = 5), WT CLP (*n* = 9), *Nlrp3* KO sham (*n* = 5), and *Nlrp3* KO CLP (*n* = 6) mice. mRNA expression was normalized to *Gapdh*. **e**, **f** Weights of the skeletal muscles, **e** *gastrocnemius/plantaris* (GP), and **f** *tibialis anterior* (TA) determined at 96 h after surgery. CLP treated *Nlrp3* KO (*n* = 16); sham *Nlrp3* KO (*n* = 8), WT CLP (*n* = 12), WT sham (*n* = 13). All weights were normalized to tibia length and expressed as percent-wise change compared to the respective sham group. Data are presented as the mean ± SEM. *p ≤ 0.05; **p ≤ 0.01; ***p ≤ 0.001; *ns* = not significant

At baseline, *Nlrp3* KO were indistinguishable from WT mice and did not differ in body, liver, spleen, or skeletal muscle weights normalized to tibia length (Additional file 3A–C). To investigate whether or not Nlrp3 inflammasome-dependent IL-1β activation affects inflammation-induced atrophy, we subjected male *Nlrp3* KO and WT mice to CLP (*Nlrp3* KO, *n* = 27; WT, *n* = 33) or sham surgery (*Nlrp3* KO, *n* = 11; WT, *n* = 16), respectively. Compared to WT mice, significantly less *Nlrp3* KO mice died after 96 h after CLP surgery (57.6 vs. 29.6%; *p* < 0.05) (Fig. 3a). Septic WT mice showed a reduction in body and liver weight and no change in spleen weight (Fig. 3b; Additional file 4A, B). In contrast, *Nlrp3* KO did not lose body or liver weight, while spleen weight increased in these mice during sepsis (Fig. 3b; Additional file 4A, B). We investigated if absence of *Nlrp3* affects muscular cytokine expression in sepsis. At baseline, *Il6* expression was not different between *Nlrp3* KO and WT in *gastrocnemius/plantaris* and *tibialis anterior* (Additional file 5A). CLP did not or only marginally induce muscular *Il6* expression in *Nlrp3* KO compared to WT (Fig. 3c, d). Also, *Il1b* expression was not different between *Nlrp3* KO and WT at baseline, and its expression was blunted in muscles of CLP-treated *Nlrp3* KO compared to WT mice (Additional file 6A, B). CLP induced *Nlrp3* expression in WT but not in *Nlrp3* KO (Additional file 6C, D). Since conversion of pro-IL-1β to IL-1β depends on an intact Nlrp3 inflammasome [14, 24, 25], we quantitated IL-1β cytokine levels in the serum of *Nlrp3* KO and WT. Already at baseline, IL-1β serum levels were reduced in *Nlrp3* KO compared to WT (Additional file 6E). CLP significantly induced IL-1β serum levels in WT. This induction in IL-1β serum levels was greatly reduced in *Nlrp3* KO (Additional file 6F). These data indicate that in sepsis muscular *Il1b* and *Il6* expression depend on Nlrp3.

**Fig. 4:**
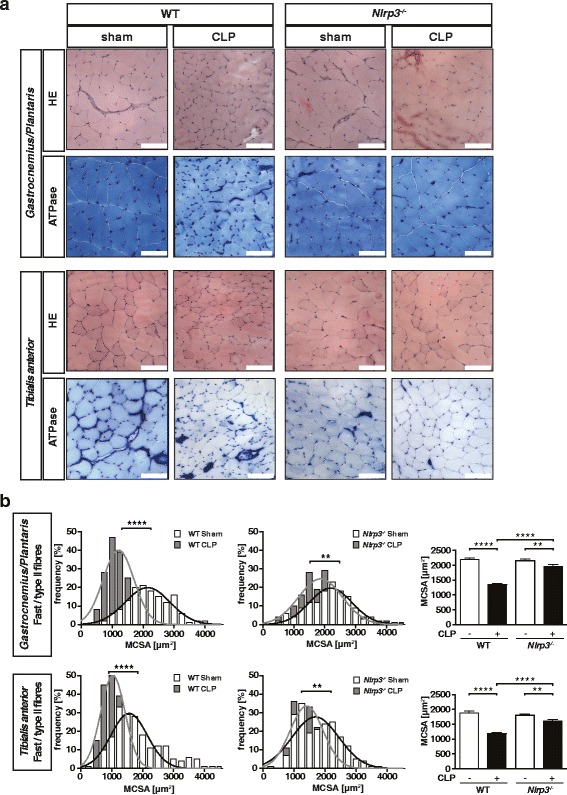
*Nlrp3* KO mice are protected from inflammation-induced muscle atrophy. 12–16-week-old male *Nlrp3* KO and WT mice were subjected to CLP or sham surgery. **a** H&E and ATPase stain of histological cross sections from *gastrocnemius/plantaris* (GP) and *tibialis anterior* (TA) muscle from sham and CLP-operated WT and *Nlrp3* KO mice at 96 h after surgery, as indicated. *Scale bar* = 100 μm. **b** Quantification of fast/type II myofiber cross-sectional area (MCSA) in GP and TA from transverse sections stained with metachromatic ATPase dye assay (as shown in **a**). MCSA was determined by using ImageJ software. *p ≤ 0.05; **p ≤ 0.01; ***p ≤ 0.001; ****p ≤ 0.0001

Compared to WT sham, WT CLP mice showed a significant reduction in the weights of all muscles investigated 96 h after surgery. In contrast, the reduction of muscle mass of *Nlrp3* KO CLP compared to *Nlrp3* KO sham mice was less severe (Fig. 3e, f, Additional file 4C, D). These data indicate that *Nlrp3* KO mice are protected against inflammation-induced atrophy. Since inflammation-induced atrophy predominantly affects fast-twitch fibers in critically ill patients [10], we analyzed atrophy of fast/type II fibers in *gastrocnemius/plantaris *and *tibialis anterior* muscles of *Nlrp3* KO and WT after CLP. The histological pictures show that myofibers of WT but not of *Nlrp3* KO CLP atrophied during sepsis (Fig. 4a). Accordingly, frequency distribution histograms of the myocyte cross sectional areas (MCSA) showed an increased number of smaller fast/type II fibers in both muscles of septic WT but not *Nlrp3* KO leading to a leftward shift of the distribution curve (Fig. 4b). This atrophic response was attenuated in *Nlrp3* KO compared to WT following sepsis as indicated by a less pronounced reduction in mean MCSA in septic *Nlrp3* KO (Fig. 4b). These data indicate *Nlrp3* contributes to fast/type II fiber atrophy in sepsis.

**Fig. 5:**
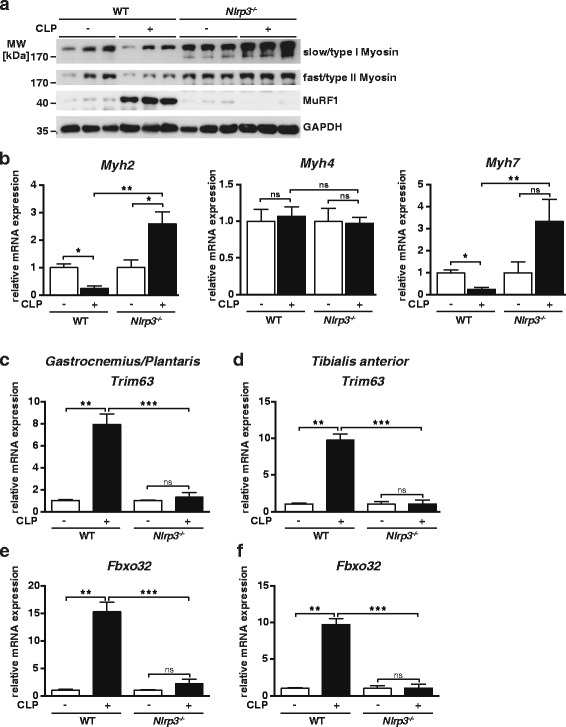
Disturbed muscular protein homeostasis in sepsis is blunted in *Nlrp3* KO mice. Twelve- to 16-week-old male *Nlrp3* KO and WT mice were subjected to CLP or sham surgery. **a** Western blot analysis with anti-myosin heavy chain (MyHC) slow and anti-MyHC fast antibody and anti-MuRF1 antibody GAPDH was used as loading control; *n* = 3. **b** qRT-PCR analysis of myosin heavy chain (*Myh*) 2, *Myh4*, and *Myh7* expression in g*astrocnemius/plantaris* (GP) muscles of sham and CLP mice at 96 h after surgery as indicated (WT sham: *n* = 5; WT CLP: *n* = 9; *Nlrp3* KO sham *n* = 5; *Nlrp3* KO: CLP *n* = 6). mRNA expression was normalized to *Gapdh*. (**c**–**f**) qRT-PCR analysis of *Trim63* (MuRF1) and *Fbxo32* (atrogin 1) expression in *gastrocnemius/plantaris* and *tibialis anterior* muscle of sham and CLP-operated WT and *Nlrp3* KO mice at 96 h after surgery as indicated (WT sham: *n* = 5; WT CLP: *n* = 9; *Nlrp3* KO sham *n* = 5; *Nlrp3* KO: CLP *n* = 6). mRNA expression was normalized to *Gapdh*. Data are presented as mean ± SEM. *ns* = not significant; *p ≤ 0.05; **p ≤ 0.01; ***p ≤ 0.001

To test if inflammatory atrophy was accompanied by a reduction in MyHC, Western blot analysis was performed and showed that sepsis caused a reduction of slow and fast MyHC protein in *gastrocnemius/plantaris* and *tibialis anterior* of WT but not *Nlrp3* KO (Fig. 5a). To elucidate if decreased myosin content was due to a reduction in MyHC gene expression, we quantitated *Myh2, 4 and 7* by qRT-PCR. Inflammation caused a significant reduction in *Myh2* and *Myh7* gene expression in WT *gastrocnemius/plantaris* muscle (Fig. 5b). In contrast, *Myh2* and *Myh7* expression increased in *Nlrp3* KO *gastrocnemius/plantaris* muscle during inflammation (Fig. 5b). Inflammation did not affect *Myh4* gene expression. In the *tibialis anterior* muscle, *Myh2* gene expression was regulated with the same trend as in *gastrocnemius/plantaris*, whereas *Myh4* and *Myh7* did not show a significant regulation (Additional file 7). To test if decreased myosin content was correlated with increased atrogene expression, we quantitated *Trim63* and *Fbxo32* expression in the muscle. Indeed, *Trim63* and *Fbxo32* expression were significantly increased in septic WT but remained unchanged in *Nlrp3* KO muscles (Fig. 5c–f). Western blot analysis showed that MuRF1 protein expression was increased in *gastrocnemius/plantaris* of WT CLP but not in *Nlrp3* KO CLP (Fig. 5a). These data indicate that decreased *Myh* gene expression and its increased degradation contribute to inflammation-induced atrophy.

## Discussion

ICUAW is a devastating disease warranting detailed mechanistic investigation [2, 45]. In our study, we found a close relationship between systemic inflammation in sepsis and muscle atrophy. We show that IL-1β signaling is present in myocytes and when activated leads to myocyte atrophy in vitro. Germline deletion of *Nlrp3* in mice led to reduced IL-1β serum levels in response to inflammation and less inflammation-induced muscle atrophy in vivo. These data underscore the conclusion that during inflammation, skeletal muscle and myocytes are targeted by IL-1β to undergo atrophy. However, since Nlrp3 is ubiquitously expressed [31, 32], the observed reduction in IL-1β serum levels in response to inflammation was most likely caused by the absence of *Nlrp3* in multiple cells, tissues, and organs and not only muscle. Nevertheless, our findings suggest Nlrp3 as target for treatment against inflammation-induced atrophy.

Based on earlier published work, we suggest that muscle contributes to inflammation and acute-phase response. Of note, the acute-phase response protein serum amyloid A1 (SAA1) was shown to be synthesized by and released from muscle of critically ill patients and septic mice [36]. SAA1 induces IL-1β expression [46] and secretion [47] and activates the Nlrp3 inflammasome in immune cells [46]. The Nlrp3 inflammasome is contained and active in C2C12 myocytes [48]*.* IL-1β induces atrogene expression in myocytes [23]. Together, these data suggest feedback loops between IL-1β, IL6, SAA1, and Nlrp3 during inflammation reinforcing muscle atrophy in critical illness. Here, we show that *Il1b* and *Il6* as well as *Nlrp3* expression are increased in muscles of septic mice. We show that the IL-1β signaling pathway is contained and active in myocytes in vitro. Based on these observations, it is possible that *Nlrp3* KO mice have less overall inflammation during sepsis when compared to *Nlrp3* WT animals and that not only decreased IL-1β levels but also reduced overall inflammation contributed to reduced muscle atrophy in septic *Nlrp3* KO mice. However, since we did not perform a comprehensive analysis of inflammation in our mice, we cannot provide a definitive answer to this hypothesis.

We hypothesized that activation of the Nlrp3 inflammasome plays a role in inflammation-mediated muscle atrophy via activation of IL-1β. The Nlrp3 inflammasome is activated by pathogen-associated molecular patterns [49] and host-derived molecules, such as DNA, which indicates cellular damage and cell death, so-called damage-associated molecular patterns [50]. Nlrp3 inflammasome could therefore contribute to both pathogen-associated immune responses and sterile-inflammation. We found that depletion of *Nlrp3* in mice not only increases their survival in sepsis but also inhibits sepsis and inflammation-mediated muscle atrophy. We believe that this phenotype is predominantly caused by the missing conversion of pro-IL-1β to IL-1β. We demonstrate that IL-1β induces atrophy presumably via the IL-1 signaling pathway leading to NF-κB activation and increased MuRF1 and atrogin 1 expression in vitro. This interpretation is in line with published work showing that IL-1β increases atrogene expression in vitro [23]. Likewise, decreased activation of IL-1β in *Nlrp3* KO mice during sepsis resulted in decreased *Il6* expression, which is a target of IL-1β and mediates atrophy [36, 51]. We hypothesize that blockage of Nlrp3 inhibits sensing of pathogen and host signals, which is followed by inhibition of IL-1β- and IL-6-dependent damage pathways resulting in better survival and reduced muscle atrophy.

IL-6 and SAA1 mRNA and protein expression are increased in muscle of critically ill patients [7, 36]. These factors promote continuous inflammation and acute-phase response that in turn triggers ICUAW. We observed that IL-1β induces *Il6* and *Nlrp3* expression in cultured myocytes. After 24 h of treatment, expression levels of both genes dropped. In contrast, in septic mice, the muscular expression of *Il6* mRNA is still markedly increased after 96 h post CLP, reflecting the situation of critically ill patients. Our data add IL-1β and *Nlrp3* as further cytokine network factors, highly expressed in the skeletal muscle during systemic inflammation. The observed differences between cultured myocytes and muscle tissue could be explained by the fact that a functional cytokine network relies on interacting organs rather than cell-to-cell communications within an isolated organ. This interpretation implies that the muscle, although itself an immune organ, requires interaction and feedback from other organs to fully respond to systemic inflammation. Taken together, during systemic inflammation, muscular expression of SAA1, IL-6, Nlrp3, and IL-1β is persistently elevated, which might contribute to atrophy.

An imbalanced muscular protein homeostasis plays a dominant role in muscle failure of critically ill patients [4, 10, 38]. Atrogin 1 and MuRF1 are key “atrogenes” in this process [10, 22, 38, 52]. Our finding that atrogene expression was not increased in *Nlrp3* KO indicates that atrogene expression is regulated by Nlrp3-dependent IL-1β activation. Muscle atrophy is accompanied by increased MyHC degradation and decreased MyHC expression [10]. Whereas sepsis led to a decreased *Myh2* and *Myh7* expression in muscle of WT, this effect was blunted in *Nlrp3* KO. Because IL-1β treatment leads to decreased *Myh2*, *Myh4*, and *Myh7* expression in myocytes, reduced muscular MyHC expression during sepsis might be caused by IL-1β. Our data indicate that Nlrp3-mediated IL-1β activation may affect both major branches of protein homeostasis and therefore regulate MyHC synthesis via mRNA expression as well as UPS-mediated MyHC degradation.

## Conclusions

We suggest that Nlrp3-mediated IL-1β activation in sepsis is a major pathogenic mechanism in inflammatory muscle atrophy. Inhibition of IL-1β could be useful to prevent ICUAW in critically ill patients. Since not only sepsis is associated with inflammation-induced muscle failure and increased IL-1β levels other forms of muscle atrophy might also profit from Nlrp3/IL-1β inhibition; for example, in patients with rheumatoid arthritis and inflammatory bowel disease. However, IL-1β is only one of many cytokines that are elevated during the cytokine storm in the acute phase of sepsis. It was shown that IL-1β, TNF-α, and IFN-γ were below detection limit in patients with septic shock admitted to an ICU [41]. In these patients, continuously high IL-6 serum levels were measured. IL-6 was not only negatively associated with muscular myosin contents, a marker for muscle atrophy, but also provoked atrophy in myocytes in vitro [41]. These data indicate that inhibition of multiple cytokines as well as its correct timing is important to reduce inflammation-induced atrophy. However, unlike during controlled conditions in animal experiments, the precise time point of sepsis onset in patients is often unknown which impedes such treatment decisions for the caring clinician. The key to a targeted therapy of inflammation-induced atrophy in sepsis is, therefore, a better characterization of the disease process, and we think that animal models are helpful in this regard.

## Additional files

Additional file 1: Supplementary Materials and Methods. (DOCX 78 kb)

Additional file 2: Polymicrobial sepsis increases *Nlrp3*, *Il1b*, and *Il6* expression in the muscle. Twelve-week-old male C57B16/J mice were subjected to cecal ligation and puncture (CLP) or sham surgery (sham), as indicated. (A) qRT-PCR analysis of *Nlrp3*, *IL-1β*, and *Il6* expression in *gastrocnemius/plantaris* (GP) and (B) *tibialis anterior* (TA) muscles of sham (*n* = 5) and CLP (*n* = 5) mice 4 days after surgery. mRNA expression was normalized to *Gapdh*. Data are presented as mean ± SEM. *p < 0.05, **p < 0.01, ***p < 0.001. (EPS 1161 kb)

Additional file 3: Body and organ weights of *Nlrp3* KO and WT mice were indistinguishable at baseline. Weights of body (A), liver and spleen (B), and muscle (C); *gastrocnemius/plantaris* (GP), *tibialis anterior* (TA), *soleus* (Sol) and *extensor digitorum longus* (EDL)); *Nlrp3* KO (*n* = 6); WT (*n* = 6). All weights were normalized to tibia length and expressed as percent wise change compared to WT. Animals were 12–16-week-old males. Data are presented as the mean ± SEM. ns = not significant. (EPS 1175 kb)

Additional file 4: Septic *Nlrp3* KO mice show no decrease in liver weight but an increase in spleen weight. Twelve- to 16-week-old male *Nlrp3* KO and WT mice were subjected to CLP or sham surgery. Weights were determined at 96 h after surgery. (A) Liver and (B) spleen weight. (C, D) Weights of the skeletal muscles (C) *soleus* (Sol) and (D) *extensor digitorum longus* (EDL) (WT sham (*n* = 13), WT CLP (*n* = 12), *Nlrp3* KO sham (*n* = 8), *Nlrp3* KO CLP (*n* = 16)). All weights were normalized to tibia length and expressed as percent wise change compared to the respective sham group. Data are presented as the mean ± SEM. ns = not significant. *p < 0.05; **p < 0.01; ***p < 0.001. (EPS 1427 kb)

Additional file 5: Baseline muscular *Il6* and *Il1b* expression in *Nlrp3* KO and WT mice. qRT-PCR analysis of (A) *Il6* and (B) *IL1b* expression in *gastrocnemius/plantaris* (GP) and *tibialis anterior* (TA) muscles as indicated. *Nlrp3* KO (*n* = 6) and WT (*n* = 6). mRNA expression was normalized to *Gapdh*. Data are presented as the mean ± SEM. ns = not significant. (EPS 1144 kb)

Additional file 6: Inflammation-induced increase in muscular *Il6* and *Il1b* expression as well as serum IL-1β levels are blunted in *Nlrp3* KO mice. Twelve- to 16-week-old male *Nlrp3* KO and WT mice were subjected to CLP or sham surgery, as indicated. At 96 h after surgery, analyses were performed. (A–D) qRT-PCR analysis of (A, B) *Il1b* and (C, D) *Nlrp3* expression in g*astrocnemius/plantaris* (GP) and *tibialis anterior* (TA) muscles of WT sham (*n* = 5), WT CLP (*n* = 9), *Nlrp3* KO sham (*n* = 5), and *Nlrp3* KO CLP (*n* = 6) mice. mRNA expression was normalized to *Gapdh*. (E, F) Serum IL-1β was determined in WT and *Nlrp3* KO mice using the Abcam kit according to the manufacturer’s protocol. (E) Serum IL-1β concentration at baseline. (F) Serum IL-1β concentration in Sham and CLP mice. WT-sham (*n* = 15), WT-CLP (*n* = 12), *Nlrp3* KO-sham (*n* = 14), *Nlrp3* KO-CLP (*n* = 14). Data are presented as mean ± SEM. ns, not significant; **p ≤ 0.01; ***p ≤ 0.001. n.d. = not detected. (EPS 1443 kb)

Additional file 7: Inflammation-induced decrease of myosin heavy chain gene expression is blunted in *Nlrp3* KO mice. Twelve- to 16-week-old male *Nlrp3* KO and WT mice were subjected to CLP or sham surgery. qRT-PCR analysis of myosin heavy chain (*Myh*) 2, *Myh4*, and *Myh7* expression in *tibialis anterior* (TA) muscles of sham (*n* = 5) and CLP (*n* = 5) mice at 96 h after surgery as indicated (WT sham: *n* = 5; WT CLP: *n* = 9; *Nlrp3* KO sham *n* = 5; *Nlrp3* KO: CLP *n* = 6). mRNA expression was normalized to *Gapdh*. Data are presented as mean ± SEM. ns, not significant; **p ≤ 0.01. (EPS 1369 kb)

## Abbreviations

CLP: Cecal ligation and puncture model of polymicrobial sepsis
Fbxo32: F-box only protein 32 (mouse gene encoding atrogin 1)
ICU: Intensive care unit
ICUAW: Intensive care unit (ICU)-acquired weakness
IL: Interleukin
MCSA: Myocyte cross-sectional area
MuRF: Muscle really interesting new gene (RING)-finger containing protein
Myh: Myosin heavy chain
Nlrp3: Nucleotide-binding oligomerization domain (NOD), leucine rich repeat and pyrin domain containing 3 (mouse gene encoding Nalp3)
qRT-PCR: Quantitative real-time polymerase chain reaction
Trim63: Tripartite motif containing 63 (mouse gene encoding MuRF1)
