# Supplementary material for: Deletion of Nlrp3 protects from inflammation-induced skeletal muscle atrophy
Source: Intensive Care Med Exp. 2017 Jan 17;5:3. doi: 10.1186/s40635-016-0115-0 (PMC5241267; doi:10.1186/s40635-016-0115-0)
Supplement: Supplementary file 1 — Supplementary Materials and Methods. (DOCX 78 kb) [file 40635_2016_115_MOESM1_ESM.docx]

**Supplementary Materials and Methods**

**Animal model.**

All animal procedures were performed in accordance with the guidelines of the Max-Delbrück Center for Molecular Medicine and the Charité-Universitätsmedizin Berlin, and were approved by the Landesamt für Gesundheit und Soziales (LaGeSo, Berlin, Germany) for the use of laboratory animals (permit numbers G 207/13, G 129/12) and followed the “Principles of Laboratory Animal Care” (NIH publication No. 86-23, revised 1985), as well as the current version of German Law on the Protection of Animals.

Polymicrobial sepsis was induced by cecal ligation and puncture (CLP) as recently reported [[1-3](#_ENREF_1)]. CLP was performed on 12-16 weeks-old male *Nlrp3* KO mice and wildtype littermates. Sequences of primers used for genotyping of mice are provided in Additional file 2. Mice were anesthetized with isoflurane, placed on a heating pad to assure a constant body temperature at 37°C measured by a rectal probe. After shaving and disinfection of the skin, midline laparotomy was performed, and the cecum was exposed and ligated using a non-absorbable surgical suture (Ethicon 6-0). A 21-gauge needle was used to puncture the cecum once, and a small amount of feces was extruded. The cecum was then replaced into the abdominal cavity, and the incisions of the peritoneum and skin were closed with two separate layers of a surgical suture (Ethicon 6-0). Sham mice were treated identically except for the ligation and puncture of the cecum. All mice were resuscitated by an intraperitoneal injection of 1 ml sterile and 37°C pre-warmed Ringer’s solution. Metamizole (200 mg/kg body weight) applied twice daily subcutaneous was used as analgesic. CLP and sham mice were sacrificed 96 h after surgery and gastrocnemius/plantaris, tibialis anterior, soleus and extensor digitorum longus were harvested for analysis. The weight of skeletal muscles were determined and tibia length was measured for reference purpose. Relative muscle weight and body weight was determined by calculating the ratio between muscle weight in [mg] and the body weight in [g], respectively, by the tibia length in [mm]. For histology gastrocnemius/plantaris, tibialis anterior, soleus and extensor digitorum longus were dissected, mounted and frozen under cryoprotection. Muscle samples were stored at -80°C. Frozen sections with a thickness of 6 µm were cut using a Leica cryotome.

**Measurement of serum IL-1β**.

ELISA was performed using the Mouse ELISA Kit for IL-1β (Abcam, ab100704) according to the manufacturers’ protocol.

**RNA isolation, cDNA synthesis and quantitative real-time-PCR**.

Total RNA was isolated from skeletal muscle biopsies or cultured cells using TRIzol® Reagent (Invitrogen) according to the manufacturer’s protocol and as recently reported [6-8]. cDNA synthesis of 1 µg of RNA per sample was carried out by using the SuperScript® First-Strand Synthesis System (Invitrogen). Quantitative real-time polymerase chain reaction (PCR) was performed using Power SYBR® Green PCR Master Mix (Applied Biosystems) and self-designed primers (for primer sequences see Additional file 3). PCR reactions were performed in a Step-One^TM^ Plus thermocycler (Applied Biosystems) as described recently using a cDNA standard curve [[1](#_ENREF_1), [4-6](#_ENREF_4)]. To correct for potential variances between samples regarding differences in mRNA extraction and reverse transcription efficiency, gene expression was normalized to the expression of the reference gene glyceraldehyde-3-phosphate dehydrogenase (*Gapdh*) [[1](#_ENREF_1), [4-6](#_ENREF_4)]. We have evaluated this housekeeping gene in our model, and found that it has stable expression across the conditions and time periods analyzed.

**Myoblast culture and differentiation**.

Cell culture experiments of murine myoblasts (C2C12 cells) were performed as described recently [[1](#_ENREF_1), [7](#_ENREF_7)]. Differentiation of myoblasts to myotubes was induced at confluence by replacing growth medium (DME medium (1 g/l glucose) (Sigma Aldrich), 10 % fetal bovine serum (FBS) (PAA), 2 mM L-Glutamine (PAA), supplemented with Penicillin and Streptomycin (both PAA)), with differentiation medium (DMEM (1 g/l glucose), 2 % FBS, supplemented with Penicillin and Streptomycin (both PAA)). Transfection of C2C12 cells was performed using Lipofectamine™ and PLUS™ reagent (both Invitrogen).

**Immunostaining of myoblasts and myotubes in vitro**.

C2C12 myoblasts and differentiated myotubes were fixed with 4 % paraformaldehyde/PBS, permeabilized with 0.2 % Triton-X-100/PBS, and blocked with 2 % goat serum/PBS. IRAK-1 was detected using anti- IRAK-1 (1:100, Calbiochem) as primary and Alexa Fluor® 488 Goat Anti-Rabbit IgG (H+L) (1:500, Invitrogen) as secondary antibody. Stained cells were embedded in ProLong Gold® Antifade Reagent that contained DAPI for nuclei stain (Invitrogen). Immunostaining was analyzed using the Leica CTR 6500 microscope and the Leica DFC 360 FX digital camera.

**Measurement of myotube diameter**.

C2C12 myotubes were treated with human recombinant IL-1β (10 ng/ml, 20 ng/ml and 50 ng/ml), dexamethasone (Dexa, 10 µM), and their respective controls for 72 h. Light microscopic pictures were analyzed using the Leica CTR 6500 microscope and the Leica DFC 360 FX digital camera. From 100 myotubes per condition three diameters per myotube were measured and averaged using the ImageJ software. The person who performed these measurements was blinded to the specific treatment of the myotubes.

**Protein extraction and immunoblotting**.

Protein analyses were performed as recently published [6, 12]. Shortly, cells were lysed in ice-cold extraction buffer (10 mM Tris HCl, pH 7.5, 140 mM NaCl, 1 mM EDTA, 25 % glycerol, 0.5 % sodium dodecyl sulfate (SDS), 0.5 % Nonident P-40, 0.1 mM dithiothreitol, 0.5 mM phenylmethylsulfonyl fluoride, 100 ng/ml protease inhibitor cocktail) and then cleared by centrifugation (4°C, 10 min, 12,000 x g). The supernatant was assayed for protein concentration using the Bio-Rad Protein Assay, frozen and stored at -80°C until usage. Protein (20 μg) was separated by 10 % SDS polyacrylamide gel electrophoresis (SDS-PAGE) and blotted onto PVDF membranes (Amersham Pharmacia Biotech). Membranes were incubated with specific primary antibodies: anti-glyceraldehyde-3-phosphate dehydrogenase (clone 6C5, Millipore, 1:20,000), anti-MuRF-1 (described earlier [[3](#_ENREF_3)], 1:1000), anti-Fast Myosin (clone My32, Sigma, 1:1000), anti-slow Myosin (clone NOQ7, Sigma, 1:1000), and secondary horseradish peroxidase (HRP) conjugated antibodies: anti-mouse IgG (Cell Signaling, 1:5000), anti-chicken IgG (Abcam, 1:2000 - 1:5000) and the signal was visualized with SuperSignal™ West Pico Chemiluminescent Substrate (Thermo Scientific). The anti-MuRF1 antibody was kindly provided by Siegfried Labeit and has been described earlier [[3](#_ENREF_3), [8](#_ENREF_8)]

**Histological analyses**.

Soleus, Extensor digitorum longus, Gastrocnemius/Plantaris, and Tibialis anterior were isolated from sham and CLP treated *Nlrp3* KO and wildtype mice after 72 h and either flash frozen in gum tragacanth (Sigma-Aldrich Chemie GmbH, Germany) or fixed in 4% paraformaldehyde and processed for routine paraffin histology as described previously [[5](#_ENREF_5)]. Frozen sections were cut on a cryotome (Leica CM 3050 S, Leica Microsystems GmbH, Germany) and stained with hematoxylin and eosin or metachromatic ATPase dye as previously described [[7](#_ENREF_7), [9](#_ENREF_9)]. ATPase staining allows identification of individual fiber types; slow/type I fibers stain dark blue and fast/type II fibers stain lighter blue. Images were acquired with a Leica CTR 6500 HS microscope, and Leica digital camera DFC 425 for histological analyses or the Leica digital camera DFC 360 Fx for fluorescence pictures (Leica Microsystems GmbH, Germany). Analyses of images were performed with ImageJ software 1.42c (http://rsb.info.nih.gov/ij/). Myocyte cross sectional area (MCSA) of type II/fast-twitch fibers were measured from metachromatic ATPase dye stained gastrocnemius/plantaris and tibialis anterior from at least 130 myofibers per genotype and condition. The person who performed MCSA measurements was blinded to the genotype and condition the animal was exposed to during the experiment.

**Luciferase reporter assays**.

C2C12 cells and HEK293 cells were maintained in DME medium (4.5 g/l glucose) supplemented with 2 mM L-glutamine, 10 % FBS, Penicillin and Streptomycin. C2C12 cells and HEK293 cells were transfected with NF-κB luciferase reporter (pGL3 basic containing three NF-κB-consensus binding sites (NF-κB-Luc), 100 ng), using 2.4 µg/ml Polyethylenimine (Polysciences, linear MW 2,500) for 48 h. To control for transfection efficacy pCMV lacZ (Clontech, 50 ng) was used in each sample. Cells were treated with recombinant IL-1β (10 ng/ml) and vehicle for 24 h, respectively, and luciferase activity was determined using Dual-Luciferase® Reporter Assay System (Promega) and normalized to fluorescence determined using FluoReporter® lacZ/Galactosidase Quantitation Kit (Invitrogen). Luciferase-to fluorescence ratios were normalized to pGL3 basic expression plasmid.

**Additional files legends**

**Additional file 4. Polymicrobial sepsis increases *Nlrp3*, *Il1b* and *Il6* expression in muscle.** 12-week-old male C57B16/J mice were subjected to cecal ligation and puncture (CLP) or sham surgery (Sham), as indicated. (**A**) qRT-PCR analysis of *Nlrp3, IL-1β* and *Il6* expression in *Gastrocnemius/Plantaris* (GP) and (**B**) *Tibialis anterior* (TA) muscles of sham (n=5) and CLP (n=5) mice 4 days after surgery. mRNA expression was normalized to *Gapdh*. Data are presented as mean ± SEM. *p < 0.05, **p < 0.01, ***p < 0.001.

**Additional file 5. Body and organ weights of *Nlrp3* KO and WT mice were indistinguishable at baseline.** Weights of body (**A**), liver and spleen (**B**), and muscle (**C**); *Gastrocnemius/Plantaris* (GP), *Tibialis anterior* (TA), *Soleus* (Sol) and *Extensor digitorum longus* (EDL)); *Nlrp3* KO (n=6); WT (n=6). All weights were normalized to tibia length and expressed as percent wise change compared to WT. Animals were 12-16-week-old males. Data are presented as the mean ± SEM. ns = not significant.

**Additional file 6. Septic *Nlrp3* KO mice show no decrease in liver weight but an increase in spleen weight.** 12-16-week-old male *Nlrp3* KO and WT mice were subjected to CLP or sham surgery. Weights were determined at 96 h after surgery. (**A**) Liver and (**B**) Spleen weight. (**C**, **D**) Weights of the skeletal muscles (**C**) *soleus* (Sol) and (**D**) *Extensor digitorum longus* (EDL) (WT sham (n=13), WT CLP (n=12), *Nlrp3* KO sham (n=8), *Nlrp3* KO CLP (n=16)). All weights were normalized to tibia length and expressed as percent wise change compared to the respective sham group. Data are presented as the mean ± SEM. ns = not significant. *p<0.05; **p<0.01; ***p<0.001.

**Additional file 7**. **Baseline muscular *Il6* and *Il1b* expression in *Nlrp3* KO and WT mice**. qRT-PCR analysis of (**A**) *Il6* and (**B**) *IL1b* expression in *Gastrocnemius/Plantaris* (GP) and tibialis anterior (TA) muscles as indicated. *Nlrp3* KO (n=6) and WT (n=6). mRNA expression was normalized to *Gapdh*. Data are presented as the mean ± SEM. ns = not significant. ns = not significant.

**Additional file 8. Inflammation-induced increase in muscular *Il6* and *Il1b* expression as well as serum IL-1β levels are blunted in *Nlrp3* KO mice.** 12-16-week-old male *Nlrp3* KO and WT mice were subjected to CLP or sham surgery, as indicated. At 96 h after surgery analyses were performed. (**A-D**) qRT-PCR analysis of (**A, B**) *Il1b* and (**C, D**) *Nlrp3* expression in G*astrocnemius/Plantaris* (GP) and *Tibialis anterior* (TA) muscles of WT sham (n=5), WT CLP (n=9), *Nlrp3* KO sham (n=5) and *Nlrp3* KO CLP (n=6) mice. mRNA expression was normalized to *Gapdh*. (**E, F**) Serum IL-1β was determined in WT and *Nlrp3* KO mice using the Abcam-kit according to the manufacturer’s protocol. (**E**) Serum IL-1β concentration at baseline. (**F**) Serum IL-1β concentration in Sham and CLP mice. WT-Sham (n=15), WT-CLP (n=12), *Nlrp3* KO-Sham (n=14), *Nlrp3* KO-CLP (n=14). Data are presented as mean ± SEM. ns, not significant; **p≤0.01; ***p≤0.001. n.d. = not detected.

**Additional file 9. Inflammation-induced decrease of myosin heavy chain gene expression is blunted in *Nlrp3* KO mice.** 12-16-week-old male *Nlrp3* KO and WT mice were subjected to CLP or sham surgery. qRT-PCR analysis of myosin heavy chain (*Myh*) 2, *Myh4* and *Myh7* expression in *tibialis anterior (TA*) muscles of sham (n=5) and CLP (n=5) mice at 96 h after surgery as indicated (WT sham: n=5; WT CLP: n=9; *Nlrp3* KO sham n=5; *Nlrp3* KO: CLP n=6). mRNA expression was normalized to *Gapdh*. Data are presented as mean ± SEM. ns, not significant; **p≤0.01.

**References**

1. Langhans C, Weber-Carstens S, Schmidt F, Hamati J, Kny M, Zhu X, Wollersheim T, Koch S, Krebs M, Schulz H, Lodka D, Saar K, Labeit S, Spies C, Hubner N, Spranger J, Spuler S, Boschmann M, Dittmar G, Butler-Browne G, Mouly V, Fielitz J, (2014) Inflammation-induced acute phase response in skeletal muscle and critical illness myopathy. PLoS ONE 9: e92048

2. Rittirsch D, Huber-Lang MS, Flierl MA, Ward PA, (2009) Immunodesign of experimental sepsis by cecal ligation and puncture. Nat Protoc 4: 31-36

3. Schmidt F, Kny M, Zhu X, Wollersheim T, Persicke K, Langhans C, Lodka D, Kleber C, Weber-Carstens S, Fielitz J, (2014) The E3 ubiquitin ligase TRIM62 and inflammation-induced skeletal muscle atrophy. Crit Care 18: 545

4. Du Bois P, Pablo Tortola C, Lodka D, Kny M, Schmidt F, Song K, Schmidt S, Bassel-Duby R, Olson EN, Fielitz J, (2015) Angiotensin II Induces Skeletal Muscle Atrophy by Activating TFEB-Mediated MuRF1 Expression. Circ Res 117: 424-436

5. Lodka D, Pahuja A, Geers-Knörr C, Scheibe RJ, Nowak M, Hamati J, Köhncke C, Purfürst B, Kanashova T, Schmidt S, Glass DJ, Morano I, Heuser A, Kraft T, Bassel-Duby R, Olson EN, Dittmar G, Sommer T, Fielitz J, (2015) Muscle RING-finger 2 and 3 maintain striated-muscle structure and function: <i>MuRF2</i> and 3 in striated muscles. Journal of Cachexia, Sarcopenia and Muscle: n/a-n/a

6. Wollersheim T, Woehlecke J, Krebs M, Hamati J, Lodka D, Luther-Schroeder A, Langhans C, Haas K, Radtke T, Kleber C, Spies C, Labeit S, Schuelke M, Spuler S, Spranger J, Weber-Carstens S, Fielitz J, (2014) Dynamics of myosin degradation in intensive care unit-acquired weakness during severe critical illness. Intensive Care Med 40: 528-538

7. Fielitz J, Kim M-S, Shelton JM, Qi X, Hill JA, Richardson JA, Bassel-Duby R, Olson EN, (2008) Requirement of protein kinase D1 for pathological cardiac remodeling. Proc Natl Acad Sci USA 105: 3059-3063

8. Moriscot AS, Baptista IL, Bogomolovas J, Witt C, Hirner S, Granzier H, Labeit S, (2010) MuRF1 is a muscle fiber-type II associated factor and together with MuRF2 regulates type-II fiber trophicity and maintenance. Journal of structural biology 170: 344-353

9. Fielitz J, Kim M-S, Shelton JM, Latif S, Spencer JA, Glass DJ, Richardson JA, Bassel-Duby R, Olson EN, (2007) Myosin accumulation and striated muscle myopathy result from the loss of muscle RING finger 1 and 3. J Clin Invest 117: 2486-2495

**Additional file 2.** Primer pairs for genotyping of *Nlrp3* knockout and wildtype mice are shown.

| **Name** | **Sequence (5’ – 3’)** |
| --- | --- |
| Mm_*Nlrp3*_WT forward | TCA AGC TAA GAG AAC TTT CTG |
| Mm_*Nlrp3*_WT reverse | ACA CTC GTC ATC TTC AGC A |
| Mm_*Nlrp3*_KO forward | TCA AGC TAA GAG AAC TTT CTG |
| Mm_*Nlrp3*_KO reverse | AAG TCG TGC TGC TTC ATG T |

*Nlrp3* indicates NOD-like receptor family, pyrin domain containing 3; WT, wildtype; KO, knockout; Mm, Mus musculus.

**Additional file 3.** Primer pairs for quantitative real-time-PCR are shown.

| **Name** | **Sequence (5’- 3’)** |
| --- | --- |
| Mm_*Fbxo32* forward | AGT GAG GAC CGG CTA CTG TG |
| Mm_*Fbxo32* reverse | GAT CAA ACG CTT GCG AAT CT |
| Mm_*Gapdh* forward | ATG GTG AAG GTC GGT GTG A |
| Mm_*Gapdh* reverse | AAT CTC CAC TTT GCC ACT GC |
| Mm_*Il1b* forward | AGT TGA CGG ACC CCA AAA G |
| Mm_*Il1b* reverse | AGC TGG ATG CTC TCA TCA GG |
| Mm_*Il6* forward | GCT ACC AAA CTG GAT ATA ATC AGG A |
| Mm_*Il6* reverse | CCA GGT AGC TAT GGT ACT CCA GAA |
| Mm_*Myh2* forward | AAC TCC AGG CAA AAG TGA AAT C |
| Mm_*Myh2* reverse | TGG ATA GAT TTG TGT TGG ATT GTT |
| Mm_*Myh4* forward | GGG AAC ATG AAA TTC AAG CAA |
| Mm_*Myh4* reverse | ATA GGC AGC CTT GTC AGC AA |
| Mm_*Myh7* forward | CGC ATC AAG GAG CTC ACC |
| Mm_*Myh7* reverse | CTG CAG CCG CAG TAG GTT |
| Mm_*Nlrp3* forward | CCC TTG GAG ACA CAG GAC TC |
| Mm_*Nlrp3* reverse | GAG GCT GCA GTT GTC TAA TTC C |
| Mm_*Trim63* forward | CCT GCA GAG TGA CCA AGG A |
| Mm_*Trim63* reverse | GGC GTA GAG GGT GTC AAA CT |

Trim63 indicates Tripartite motif 63 (MuRF1); Fbxo32, F-Box only 32 (atrogin1); Gapdh, glyceraldehyde-3-phosphate dehydrogenase; Il6, interleukin 6; Il-1β, interleukin 1β; Myh, myosin heavy chain; Nlrp3, NOD-like receptor family, pyrin domain containing 3; Mm, Mus musculus.
